# A Combined Transcriptomics and Proteomics Approach Reveals the Differences in the Predatory and Defensive Venoms of the Molluscivorous Cone Snail *Cylinder ammiralis* (Caenogastropoda: Conidae)

**DOI:** 10.3390/toxins13090642

**Published:** 2021-09-10

**Authors:** Samuel Abalde, Sébastien Dutertre, Rafael Zardoya

**Affiliations:** 1 Departamento de Biodiversidad y Biología Evolutiva, Museo Nacional de Ciencias Naturales (MNCN-CSIC), José Gutiérrez Abascal 2, 28006 Madrid, Spain; rafaz@mncn.csic.es; 2 Department of Zoology, Swedish Museum of Natural History, Frescativägen 40, 114 18 Stockholm, Sweden; 3 IBMM, Université de Montpellier CNRS, ENSCM, 34095 Montpellier, France; sebastien.dutertre@umontpellier.fr

**Keywords:** Conidae, transcriptome, proteome, conotoxin, cono-insulin, *Cylinder ammiralis*

## Abstract

Venoms are complex mixtures of proteins that have evolved repeatedly in the animal kingdom. Cone snail venoms represent one of the best studied venom systems. In nature, this venom can be dynamically adjusted depending on its final purpose, whether to deter predators or hunt prey. Here, the transcriptome of the venom gland and the proteomes of the predation-evoked and defensive venoms of the molluscivorous cone snail *Cylinder ammiralis* were catalogued. A total of 242 venom-related transcripts were annotated. The conotoxin superfamilies presenting more different peptides were O1, O2, T, and M, which also showed high expression levels (except T). The three precursors of the J superfamily were also highly expressed. The predation-evoked and defensive venoms showed a markedly distinct profile. A total of 217 different peptides were identified, with half of them being unique to one venom. A total of 59 peptides ascribed to 23 different protein families were found to be exclusive to the predatory venom, including the cono-insulin, which was, for the first time, identified in an injected venom. A total of 43 peptides from 20 protein families were exclusive to the defensive venom. Finally, comparisons of the relative abundance (in terms of number of peptides) of the different conotoxin precursor superfamilies showed that most of them present similar abundance regardless of the diet.

## 1. Introduction

Venom systems are chemical weapons that confer a selective advantage and have evolved repeatedly in different animal lineages [1,2]. The best-characterized venom systems are arguably those of snakes [3] within vertebrates and those of cone snails [4] and spiders [5] within marine and terrestrial invertebrates, respectively. Animal venoms are complex mixtures dominated by peptides, and certain groups can dynamically adjust their venom’s composition depending on external stimuli and whether they are used to capture prey or deter predators [6]. Venoms are a rich natural source for the discovery of drugs with painkiller, anti-inflammatory, or antihypertensive properties, and thus, they have great potential for human therapeutics [7]. Hence, there is active research devoted to catalogue animal venoms.

The powerful combination of high-throughput proteomic and transcriptomic approaches has become the most efficient methodology to disentangle the highly complex mixtures of venoms and identify their components [8]. The advent of RNA sequencing [9] opened the door to identify the complete set of transcripts that are expressed in the venom gland of a venomous animal. These transcripts encode for toxins, as well as for proteins involved in the folding and maturation of the toxins [10]. Comparisons with other transcriptomes provide relevant information about the evolution and relative importance of these proteins. If comparisons involve other tissue transcriptomes, it is possible to uncover those genes that are essential for venom production, as well as variations in their expression in response to different stimuli [3]. If comparisons are between venom gland transcriptomes of related species, it is possible to discriminate those toxins that are shared-derived from common ancestors from those that are exclusive of a given species, as well as uncover instances of convergence [11]. In addition, a fraction of the venom gland transcripts is expressed in rather small quantities, whereas others may be even degraded before translation [12]. Moreover, the set of proteins that are synthesized in the venom gland do not necessarily match with the actual toxins found in the secreted mature venom, as the individual can dynamically adjust its composition depending on the stimuli [13]. Hence, it is important to also determine the venom gland proteome through mass spectrometry and compare it with the corresponding transcriptome to define the functional composition of the venom, including the identification of numerous post-translational modifications [14]. Finally, venom glands show regionalization of toxin production [6,15], which can be best characterized combining transcriptomics and proteomics approaches [16].

The more than 900 described species of cone snails live in tropical and subtropical marine waters around the world [17]. They use venom in a very versatile way, as they have diverse hunting behaviors [18]; can prey on worms, snails, or fishes [19,20]; and can also use their venom to deter predators [6]. Each species synthesizes in a specialized venom gland a complex mixture of hundreds of peptides generally known as conotoxins that block neuromuscular receptors and ion channels to produce different physiological effects, including sedation, paralysis, or even death [21]. Conotoxins are first synthesized as precursors with signal, propeptide, and mature regions. The signal region is the most conserved and is used to classify the precursors into superfamilies [21]. After cleavage, the mature region becomes the functional toxin and is often characterized by specific disulfide bonds and post-translational modifications. In addition to conotoxins, species of the genus *Gastridium*, which engulf schools of small fishes with the rostrum before injecting venom through the proboscis [18], have evolved a specialized strategy to sedate fish prey that includes an insulin mimic to cause hypoglycemic shocks [22]. Interestingly, the monomeric structure of this insulin has been used to design a small and fully active insulin analog to treat human diabetes [23]. Apart from peptides, the venom of the West African species *Genuanoconus genuanus* produces a guanine derivative, genuanine, with toxic properties, indicating that the overall complexity of cone snail venom cocktails is far from being fully described [24].

Thus far, most studies on cone snail venomics either describe the transcriptome or the proteome of a given species. The full sets of transcripts encoding conotoxin precursors have been characterized in vermivorous [25], molluscivorous [26], and piscivorous [15,27] species, showing striking differences in composition. For instance, distinct members of the A superfamily are found in the venom of piscivorous *Chelyconus ermineus* [15] and *Pionoconus magus* [27]. Similarly, the toolkits of mature peptides of species with different diets are being catalogued from proteomes (e.g., Reference [28]). In the case of cono-insulin, this hormone has been annotated in the venom gland transcriptome from several species [22,29], and the presence of the actual protein in the venom gland was later confirmed [30]. However, this protein remains to be detected in the proteome of an injected venom, confirming its utilization during prey hunt. As in the case of snakes [31], the best results are achieved by combining both approaches, and thus, studies determining simultaneously both transcriptomes and proteomes of cone snails are becoming the standard [16,32,33,34].

Here, we performed a combined transcriptomic and proteomic approach to characterize the composition of the venom produced by the admiral cone snail, *Cylinder ammiralis*, a molluscivorous species of wide distribution in the Indo-Pacific region. For the proteome investigation, we milked predation-evoked and defense-evoked venoms and compared them with the venom gland transcriptome, revealing differences in composition. Moreover, we detected, for the first time, a cono-insulin in the predation-evoked venom of a cone species and demonstrated the use of hormone-like conopeptides during prey capture.

## 2. Results

### 2.1. Composition of C. ammiralis Venom

#### 2.1.1. Venom Gland Transcriptome

The transcriptome of the venom duct from one specimen of *Cylinder ammiralis* was sequenced, generating 31.7 million reads. A total of 800,729 reads (2.52%) were removed during the cleaning step, and the remaining reads were assembled into 86,230 contigs. BLASTX (National Center for Biotechnology Information (NCBI), Bethesda, MD, USA) searches against the custom database returned 1486 hits. After removing false positives, sequences with low coverage (<5 mapped reads), assembly artifacts, truncated peptides (<45% of the length), and duplicated sequences, the transcripts of 242 conotoxin precursors, hormones, and venom-related proteins were annotated, with most of them being full-length (72.72%). The majority of the incomplete peptides lacked the N-terminus, whereas only 17 (7.02%) had no stop codon. All information related to these sequences is available in Supplementary Materials Table S1 and File S1.

**Figure 1:**
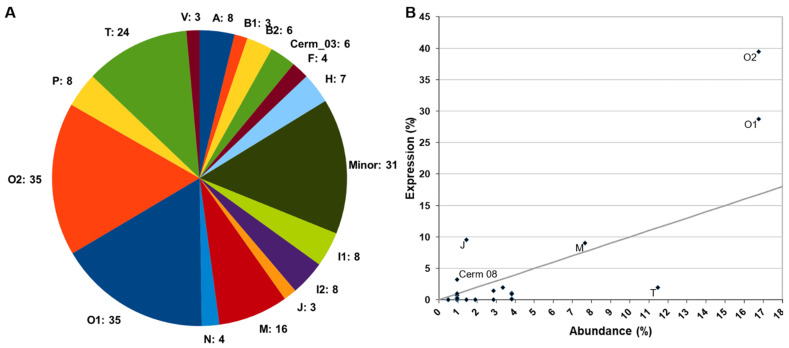
Summary of the diversity of conotoxins of *Cylinder ammiralis* based on the venom gland transcriptome. (**A**) Distribution in superfamilies of the 209 identified conotoxin precursors. The number beside the name represents the number of peptides within the superfamily. The minor superfamilies are expanded in Supplementary Materials Figure S1. (**B**) Scatter plot displaying the correlation between the number of peptides and the relative expression (measured in Transcripts Per Million, TPMs) for each superfamily. The two values were transformed to percentage. The gray line represents the 1:1 correlation between the two axes.

A total of 209 conotoxin precursors were classified into 35 superfamilies based on the similarities of their signal regions. The most abundant superfamilies (those presenting more peptides) were O1 (35 conotoxin precursors), O2 (35), T (24), and M (16), which added up more than half of all identified conotoxin precursors (52.6%; Figure 1A, expanded in Supplementary Materials Figure S1). All other superfamilies had less than ten precursors, and 24 of them were represented by less than five. Only one out of the five conotoxins (belonging to A and O1 superfamilies) previously reported for *C. ammiralis* in ConoServer (http://www.conoserver.org/, accessed on 7 August 2021) [35] was recovered in this study (Supplementary Materials File S2).

The relative expression level of each conotoxin precursor superfamily was calculated as the sum of the expressions of all its members. Three of the superfamilies with more peptides were also recovered among the most expressed (Figure 1B): O2 (39.49% of the expression), O1 (28.77%), and M (9.03%). Despite its high diversity of peptides, the T superfamily only accounted for 1.95% of the overall conotoxin precursor expression, almost at the same level of the H superfamily (1.92%, seven conotoxin precursors). The three precursors of the J superfamily represented the third highest expression level (9.57%). Cerm 08 had an intermediate expression level (3.24%), and all other conotoxin precursor superfamilies showed expression levels lower than 1.5%, with 18 of them below 0.05% (Figure 1B). A more detailed representation of these results can be found in Supplementary Materials Figure S2. The 20 most expressed superfamilies (accounting for 71% of overall conotoxin precursor expression) are shown in Supplementary Materials File S3.

The BLASTX searches identified 13 hormone-like conopeptides in the venom gland transcriptome of *C. ammiralis* (Supplementary Materials Table S1): two Conopressins, three Conorfamides, two Insulins, four Prohormone-4, and two Thyrostimulins (alpha and beta). In addition, 20 venom-related proteins were identified (Supplementary Materials Table S1), of which Ferritin (four paralogs) and Protein Disulfide Isomerase (PDI; nine paralogs) were the two most common.

#### 2.1.2. Mass Spectrometry Analysis of Predatory and Defensive Venoms

**Figure 2:**
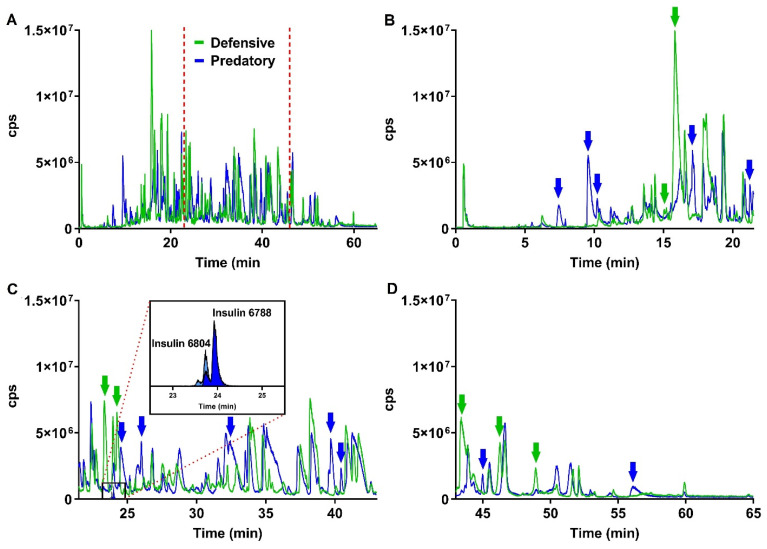
(**A**) LC–MS profiles of the predatory (blue) and defensive (green) venoms of *Cylinder ammiralis*. The red dashed lines mark the limits of the expanded sections in (**B**–**D**). The arrows highlight the main differences between the two venoms, using the same color code. In the panel (**C**), the masses of the insulin are shown in the inset.

**Figure 3:**
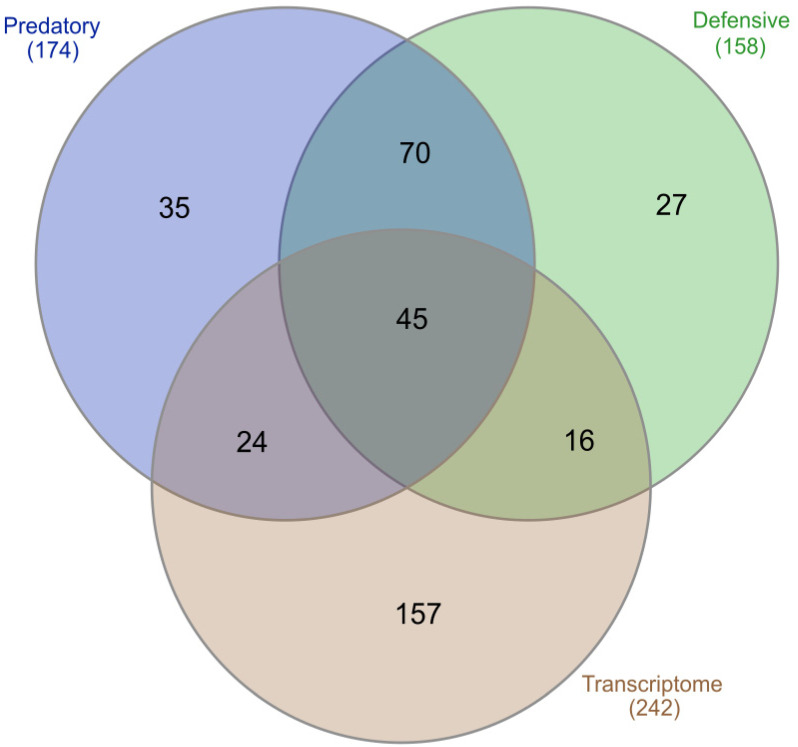
Number of unique and shared proteins found in the predatory venom (blue), defensive venom (green), and transcriptome (brown). The number under the category name represents the total number of proteins annotated for each dataset. The Venn diagram was built by using InteractiVenn (http://www.interactivenn.net, accessed on 7 August 2021) [36].

The predation- and defense-evoked venoms of *C. ammiralis* were collected separately. Both extracts showed markedly distinct yet equally complex profiles (Figure 2). A total of 217 different peptides were identified, of which only about half (115 or 53%) were common to the two venoms (Figure 3 and Supplementary Materials Table S2). Using the transcriptome as a reference, as well as blast searches against GenBank (https://www.ncbi.nlm.nih.gov/genbank/, accessed on 7 August 2021) and UniProt (https://www.uniprot.org/, accessed on 7 August 2021), all but 35 peptides could be annotated and assigned to 47 different (super)families. The ten most common protein families were M (nine peptides), O1 (nine), von Willebrand factor (eight), O2 (seven), T (seven), I1 (five), Angiotensin (four), Collagen alpha-4 (three), J (three), and PDI-A2 (three).

The predatory venom included 174 peptides, which were classified into 56 different protein families (except for 26 that remained unidentified; Figure 3 and Supplementary Materials Table S2). Conotoxins represented the main component of this venom, with 86 members. The (super)families with more peptides were O2 (20 peptides), O1 (14), M (13), T (ten), I1 (seven), and von Willebrand factor (eight). A total of 59 peptides ascribed to 23 different protein families were found to be exclusive to the predatory venom (Figure 3), including Actin, Coagulation factor, E superfamily, Elongation factor, I4 superfamily, Insulin (highlighted in Figure 2), and Synaptic vesicle membrane protein, with each one represented by one single peptide (Supplementary Materials Table S2).

Analysis of the defensive venom recovered 158 peptides, which were classified into 57 protein families (except for 23 that remained unidentified; Figure 3 and Supplementary Materials Table S2). The most common conotoxin superfamilies were O1 (14), O2 (12), M (ten), T (seven), and I1 (six). A total of 43 peptides from 20 protein families were exclusive to the defensive venom, including Calreticulin, Conodipine, FK506-binding protein, Galactose-binding, I2 superfamily, PLAT-LH2, Thioredoxin peroxidase, Tpra 06 superfamily, and U superfamily (Supplementary Materials Table S2).

#### 2.1.3. Transcriptome versus Proteomes

Of the 374 venom components annotated in this study, 85 were common to both the transcriptome and proteome approaches (Figure 3). These components included 19 conotoxin superfamilies, all the hormones, and four venom-related proteins (CAP, Condipine, Neuropeptide F, and PDI-A2; Supplementary Materials Table S2). Moreover, 132 out of the 217 peptides (61%) were not annotated in the transcriptome by using the custom database (Figure 3; Supplementary Materials Table S2). Conversely, 157 out of 242 transcripts (65%) were absent from the two proteomes (Figure 3; Supplementary Materials Table S2). The proteins annotated only in the proteome were classified into 36 different venom-related protein families. Most of the families found exclusively in the transcriptome had few members: B2 and Cerm 03 had six peptides each, and the other 12 superfamilies presented less than five peptides (Supplementary Materials Table S2). Six venom-related protein families were only found in the transcriptome, including two variants of the abundant PDI and the Ferritin (Supplementary Materials Table S2).

### 2.2. Differences in Venom Composition across Diets

**Figure 4:**
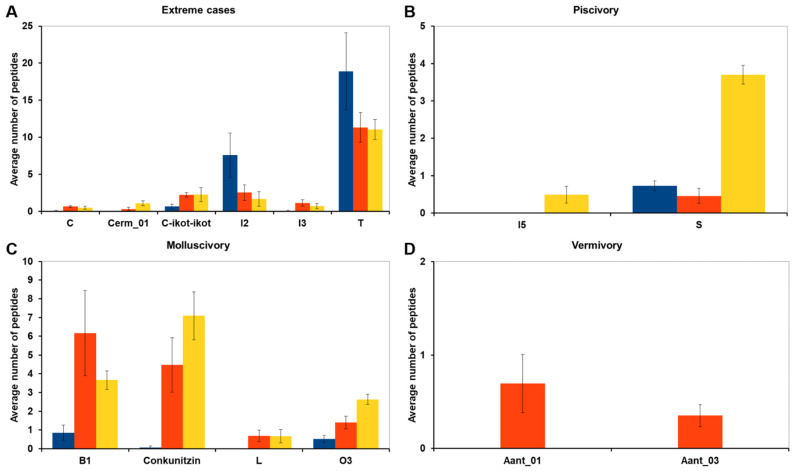
Summary of the superfamilies whose abundance is statistically different among diets. The three diets are shown in blue (molluscivory), orange (vermivory), and yellow (piscivory). The bars represent the average number of peptides per diet, and the error bars the standard error on the mean. (**A**) The differences were statistically different only between the lowest and the highest abundances. In the other three panels, the abundance of each superfamily was statistically different in one of the diets respect to the other two: (**B**) piscivory, (**C**) molluscivory, and (**D**) vermivory.

The compositions of the venom gland transcriptomes of *C. ammiralis* and other cone snails with different diets (six molluscivorous, six vermivorous, and six piscivorous) were compared, aiming at identifying conotoxin precursor superfamilies that appear significantly abundant (in terms of number of peptides) in a given diet. The comparison included 69 conotoxin precursor superfamilies, of which 55 showed no statistically significant differences between diets (Supplementary Materials Table S3). For six conotoxin precursor superfamilies, differences in abundance were significant only when the two most extreme cases were compared (i.e., the two diets with the highest and lowest number of peptides and excluding the third diet with intermediate abundance; Figure 4A and Supplementary Materials Table S3). The abundance of Cerm 01 and Con-ikot-ikot superfamilies was statistically significantly lower in molluscivorous than in piscivorous cones; the abundance of C and I3 superfamilies was statistically significantly lower in molluscivorous than in vermivorous cones; finally, the I2 and T superfamilies were significantly more abundant (in average 4.4 and 1.7 times, respectively) in molluscivorous than in piscivorous cones (Figure 4A and Supplementary Materials Table S3).

The abundance of eight conotoxin precursor superfamilies was statistically significantly different in one diet compared to the other two (Figure 4B–D). The I5 and S superfamilies showed a statistically significant higher abundance in piscivorous cones (Figure 4B). Although the differences in the I5 superfamily were subtle, as it was present only in individuals from *Pionoconus magus*, the differences in the abundance of the S superfamily were particularly relevant, presenting, on average, five times more conotoxins in piscivorous than in molluscivorous or vermivorous species (Figure 4B and Supplementary Materials Table S3). The Conkunitzin, L, and O3 superfamilies had a statistically significant lower abundance in molluscivorous cone snails (Figure 4C and Supplementary Materials Table S3). Finally, Aant 01 and Aant 03 superfamilies (unassigned superfamilies in Reference [11], and here named after *Africonus antoniomonteroi*) were statistically more abundant in vermivorous species (Figure 4D and Supplementary Materials Table S3).

Overall, molluscivory was involved in all the above-described 14 significant abundance difference comparisons. This diet presented a significantly lower number of peptides than piscivorous and vermivorous cones in 12 out of the 14 superfamilies. The abundance of the S superfamily was much lower in molluscivorous than in piscivorous cones, and just slightly higher than in the vermivorous. The only exception to this trend were the I2 and T superfamilies with higher abundances in molluscivorous species; however, the standard deviation in these cases was between one-fourth and one-third of the average number of peptides, indicating a high variability among specimens (Figure 4A and Supplementary Materials Table S3).

## 3. Discussion

### 3.1. Transcriptomics and Proteomics, Two Complementary Approaches to Determine Venom Composition

The venoms of each of the >900 species of cone snails are a rich natural source of potential medical drugs [7]. However, isolating and characterizing the functional constituents of cone snail venoms is challenging, as they are peptide cocktails of complex and dynamic composition. A combined transcriptomic and proteomic approach is currently the most efficient method to catalogue the peptides that are produced by an individual in the venom gland, as well as the fraction that is loaded into the harpoons to capture prey or deter predators [6,16]. Here, we show that results obtained by using transcriptomics and proteomics in identifying the venom toolkit of the admiral cone snail, *C. ammiralis*, are complementary. Nevertheless, there is a possibility that differences in transcriptomic and proteomic data are due to variability associated to the comparison of individuals from different locations. A total of 242 conotoxin precursors, hormones, and venom-related proteins were identified in the transcriptome of the venom gland, a number that fits well within the range reported for other species of cone snails, such as *P. magus* [27], *D. betulinus* [25], and *Varioconus guanche* [11]. Similarly, a total of 217 different peptides were identified in the predatory and defensive proteomes of *C. ammiralis*, also within the expected range [28,32].

Despite that the numbers recovered by the two approaches are very similar, only about a half of the venom components were common to both. As it has been shown in other studies, the high level of intra-specific variation makes the venoms from different individuals virtually different [15,25,37]. Hence, the differences observed between the two approaches could be attributed to the proteomes and the transcriptome coming from individuals from different localities. However, since the proteomic data were assembled and curated by using this same transcriptome as reference, all proteins annotated in the two proteomes should also be present in the transcriptome. This discrepancy might be explained by the intrinsic differences of the reference databases used to annotate the transcriptome and the proteomes. All the peptides identified in the proteome and not the transcriptome belong to venom-related proteins, with the exception of one peptide present in both the predatory and defensive venoms that belong to R superfamily, which was recently described in the vermivorous *Conasprelloides villepinii* and *Gradiconus anabathrum*, and it is hypothesized to block potassium channels [38]. Instead, the reference database used to annotate the transcriptome is mostly focused on conotoxin precursor superfamilies, several hormones, and a few known venom-related proteins, which indeed form most of the 85 proteins found in common to both approaches. The transcriptome and the proteomes shared most diverse superfamilies, such as O1, O2, T, and M. These superfamilies tend to be the majority in all species, and they have been proposed to constitute the basic toolkit of the cone snail venom; moreover, they could be already present in the ancestor of the group [11]. In *C. ammiralis*, O2, O1, and M superfamilies were highly expressed, but not the T superfamily. In contrast, the J superfamily, with only three transcripts, showed the third highest expression level. The expression of these four superfamilies is remarkable, accumulating in only 20 peptides more than 70% of the total conotoxin precursor expression, and prompting a more careful study of these peptides. In particular, the J superfamily has been reported to be widespread in vermivorous cone snails, such as *Strategoconus planorbis* and *Strategoconus ferrugineus* [39]; *Tesselliconus eburneus* [40]; *D. betulinus* [25], *Turriconus praecellens* [37]; molluscivorous cone snails, such as *C. victoriae* [41]; and in piscivorous cone snails, such as *C. ermineus* [15] and *P. magus* [27]. The conotoxins of the J superfamily are one of the few that block both voltage-gated (K^+^) and ligand-gated (nicotinic acetylcholine) ion channels [39]. The high expression level of this superfamily, its presence in both proteomes, and its widespread distribution among diets might be an indicator of the importance of this superfamily to capture prey and deter predators in this species.

The annotation of several proteins exclusively in the transcriptomic approach was not related to a particularly low expression of the corresponding transcripts, as many conform to average expression levels of the whole transcriptome, and, for instance, conotoxin precursor OK053_207, belonging to the O1 superfamily, was actually the transcript with the highest expression in the transcriptome (4.68%). In this regard, it is important to note that transcriptome assembly and annotation are not straightforward steps, and some extra transcripts may correspond to assembly artifacts or background expression of housekeeping genes [42].

Based on BLAST searches against the complete NCBI and UniProt databases, we identified up to 6964 open reading frames (ORFs) in the transcriptome of *C. ammiralis*. Apart from conotoxin precursor superfamilies, hormones, and the few known venom-related proteins, it is difficult to determine whether all other ORFs inferred in the venom gland transcriptome are venom-related or housekeeping proteins. In this regard, the list of proteins annotated in the proteome is key to identify those proteins that are functional in the secreted venom and help completing the annotation of the venom gland transcriptome. In fact, the annotation of the complete transcriptome included the identification of two-thirds of the 36 protein families annotated only in the proteome, whereas 12 of them remained elusive, stressing the importance of combining the two approaches to obtain a complete picture of venom composition [42]. In addition, future comparisons to other tissue transcriptomes could help identifying those transcripts (proteins) that are exclusively or differentially overexpressed in the venom gland.

### 3.2. Predatory- versus Defensive-Evoked Venoms

A classic histological study of the venom gland of *P. magus* already showed that the proximal and distal regions could produce venoms with different pharmacological properties [43]. More recently, the regionalization of venom production along the venom gland was associated to predatory- (distal region) and defensive-evoked (proximal region) responses in *Gastridium geographus* [6]. Here, we studied the predatory- and defensive-evoked venoms of *C. ammiralis*. Both venoms shared 115 peptides, which represent 57% of the predatory venom and 75% of the defensive venom. The O1, O2, M, and T superfamilies were the most common in the shared fraction. Another peptide that was relatively diverse in both the predatory and defensive venoms was the von Willebrand factor (VWF). The presence of this peptide is intriguing, as it is known to interact with platelets to initiate hemostatic plug formation. In the venom of snakes, C-type lectin-like proteins and Zn^2+^ metalloproteinases bind VWF and interfere with platelet aggregation [44]. The presence of neprilysin, a Zn^2+^ metalloproteinase, was also detected in both the predatory and defensive venoms.

Another protein related to hematologic disorders, the multiple coagulation factor deficiency protein 2 (MCFD2), was found exclusively in the predatory venom, suggesting that this venom might have hemorrhagic properties. All members of the A superfamily were detected in the predatory venom, but one was also found in the defensive venom. The A superfamily, together with the S superfamily, has been recognized as particularly determinant for the toxic effect of the venom of piscivorous cone snails [15,27]. One member of the E and I4 superfamilies was isolated exclusively in the predatory venom. These conotoxins were also reported in two other molluscivorous species, *C. victoriae* [41] and *C. gloriamaris* [26], although their function remains unknown. Finally, an insulin analog was isolated exclusively in the predatory venom. Although the expression of a specialized insulin in the venom duct of piscivorous *Gastridium* species was reported [22], this is the first time that the protein is isolated in a secreted venom and associated specifically to a predatory behavior. Moreover, the use of insulin was associated to the particular hunting behavior of *Gastridium* species, which engulf fishes with the rostrum and then inject the venom [18]. However, the use of insulin in a molluscivorous cone snail indicates that causing hypoglycemic shocks is a more general preying strategy.

The defensive venom had exclusively the conodipine, a type of phospholipase A2 (PLA2), which has been found in species of the piscivorous genera *Chelyconus* and *Pionoconus*, vermivorous species of the genus *Kioconus*, and the molluscivorous species *C. victoriae* (see Reference [45] and the references therein). This enzyme catalyzes the Ca^2+^-dependent hydrolysis of phospholipids, and as a component of snake venom, it has myotoxic effects and produces local pain and inflammation [46]. Another interesting enzyme exclusive of the defensive venom was the Thioredoxin peroxidase (TPX), which catalyzes the reduction of hydrogen peroxide, protecting against tissue damage, and was reported in the venom gland of honey bee workers that use venom for defense [47]. The first peptide belonging to the U superfamily was reported in *Cylinder textile,* and its injection in mice triggered jumping and convulsion [48]. It was later found in *C. victoriae* [41], *C. gloriamaris* [26], and *Leptoconus amadis* [49]. Here, we detected this superfamily only in the defensive venom of *C. ammiralis*. Its ubiquity in molluscivorous species and its presence exclusive to the defensive venom suggest the importance of this superfamily in defensive responses within this group. Nevertheless, members of this superfamily were also identified in the venom gland transcriptomes of the vermivorous Indo-Pacific *Turriconus praecellens* [37] and West African *Africonus* and *Varioconus* [11].

Although proteomic analysis of the venom gland is a common approach to the characterization of venom composition, the common practice is the dissection of the venom gland, which does not allow us to make inferences about the specific toxins used during prey hunting or defense against predators. Regarding mollusk hunter cones, there are two cases where the injected venom was studied. The injected venom of *C. textile* was analyzed, showing variation in venom composition in successive injections [50]. As in *C. ammiralis*, this venom that would constitute the predatory venom of this species was rich in M, O, and T conotoxin superfamilies. Furthermore, the predatory and defensive venoms of *Conus marmoreus* have also been described [6]. The defensive venom of *C. marmoreus* was more complex than the predatory one, contrasting with the pattern observed in *C. ammiralis*. Sadly, these two studies were mostly focused on the general profile of the venom, and little detail about their composition is provided.

The compositions of the defensive and predatory venoms of *C. ammiralis* differ considerably from those of the corresponding venoms in the piscivorous *Gastridium* [6]. In the latter, the defensive venom was mainly composed by members of the O1, M, and A superfamilies, whereas the predatory venom contained mainly contryphan and conopressin/conophysin. All of these superfamilies were common to both venoms in *C. ammiralis,* except the A superfamily, which was found almost exclusively in the predatory venom, contrary to the observation in *Gastridium* [6]. The predatory venom of *Rhombiconus imperialis*, a cone snail species that preys on fireworms (family Amphinomidae), was reported to contain mainly members of the T and K superfamilies [16]. These striking differences in the composition of predatory and defensive venoms are likely associated with the different diets, hunting behaviors, and types of predator encountered by these species, emphasizing the need to better understand the ecology of the stunning diversity of cone snails.

### 3.3. Comparative Analyses of Venom Composition across Diets

The initial characterization of venom gland transcriptomes from cone snails was based on the 454-pyrosequencing technology, which did not require assembly procedures to obtain the conotoxin precursor sequences (e.g., Reference [51]). However, this technology is now discontinued and superseded by Illumina-based RNA sequencing (Illumina, San Diego, CA, USA), which is more powerful and capable of detecting virtually all transcripts (even those with low expression) but requires an assembly step (e.g., References [15,25,26]). As a consequence, the comparison of 454-based and Illumina-based venom gland transcriptomes is not straightforward. Moreover, the rapid accumulation of newly described conotoxin precursor superfamilies implies that these might be present but not annotated in older transcriptomes. Finally, the names of the superfamilies are not always consistent across studies, and careful comparison of annotations is required. Fortunately, Illumina-based venom gland transcriptomes are rapidly accumulating, and annotations are becoming standardized, thus allowing comparative studies on the composition of venoms across species [11,19]. These studies are fundamental for understanding the adaptive value of this important functional trait and its relation with dietary specialization [19]. Here, we compared the relative abundance of the different conotoxin precursor superfamilies (in terms of number of members) in the venom gland transcriptomes of 19 cone specimens, taking into consideration their diet (worms, snails, or fishes). The relative abundance of most conotoxin precursor superfamilies (55 out of 69; 80%) was similar in the different species, regardless of the diet. Through natural selection, this master formula has been fine-tuned and adapted to specific prey and predators by developing a toxin variant capable of triggering different physiological responses, as well as modulating the abundance and expression of specific conotoxin precursor superfamilies in the different cone snail species. For instance, the S superfamily contains, on average, five times more conotoxins in piscivorous than in molluscivorous or vermivorous species. The S superfamily conotoxins have been described in many cone snail species and are involved in the inhibition of ligand-gated serotonin and nicotinic acetylcholine receptors [21]. In the piscivorous *C. ermineus*, this superfamily showed significantly higher expression in the distal region [15]. Venoms of molluscivorous species were characterized by significantly lower number of peptides than those of piscivorous and vermivorous cones in 12 out of 14 conotoxin precursor superfamilies. Only I2 and T superfamilies showed higher abundances in molluscivorous species, although standard deviations indicated high variability among specimens. The conotoxins of the T superfamily can have cysteine frameworks V and X [21]. The latter is typical of molluscivorous species, including *C. marmoreus* [40], *Conus araneosus* (Genbank entry AKJ51806), *C. victoriae* [41], and *C. ammiralis* (this work), but also found in the piscivorous *P. magus* [27]. Finally, the only two conotoxin superfamilies highlighted in vermivorous cones in respect to the other two are Aant 01 and Aant 03. However, these superfamilies have recently been described in West African cones [11] and thus far have been reported only in these species. No function is known to these conotoxins.

A principal component analysis (PCA) was used to analyze general differences in venom composition, and it showed a defined pattern between the three diets (Supplementary Materials Figure S3). Molluscivorous cones had a remarkable compact pattern, with *C. ammiralis* being the most separate species in the PCA. Similarly, piscivorous cones were recovered together, whereas the vermivorous cones were highly dispersed. Molluscivorous cones have a monophyletic origin [33], and independent origins have been postulated for Atlantic and Indo-Pacific piscivorous cones (represented by the two species here analyzed), respectively [15]. However, vermivory has been inferred to be the ancestral condition in cones and is widespread throughout the Conidae Tree of Life. Therefore, our sampling may have many gaps among vermivorous lineages, thus artificially inflating the differences within this group.

## 4. Conclusions

In this study, we characterized the venom gland transcriptome of the molluscivorous species *C. ammiralis*, together with the proteomic analysis of both its predatory and defensive venoms. Although a core of common components was identified, our results also demonstrate some purpose-specific conotoxin and venom proteins that are uniquely found in either the predatory or defensive venom. More work—particularly on the functional effects of several classes of conotoxins—is required for a better understanding of the evolution and specialization of these animals. Overall, this study highlights the necessity to combine venom gland transcriptomics and predatory and defensive venom proteomics with more ecology-based observations in order to draw meaningful conclusions on the purpose of each conotoxin type injected.

## 5. Materials and Methods

### 5.1. Taxon Sampling

For transcriptome analyses, one adult specimen of *C. ammiralis* was captured in Kyoda Bay (Okinawa island, Japan), in 2017, with corresponding permits. This individual was dissected in a resting stage to remove the venom duct, which was stored in 1 mL of RNAlater (Invitrogen, Life Technologies, San Diego, CA, USA) at 4 °C during the sampling campaign and at −20 °C for the long-term. For proteome analyses, a total of two adult specimens of *C. ammiralis* were captured from the Southern Great Barrier Reef (Queensland, Australia), under the corresponding permits, and kept alive at the University of Queensland aquarium facility, with the temperature set to 23 to 24 °C, in a 12:12 light-dark cycle.

The species here studied belongs to the genus *Leptoconus* according to the morphology-based classification of cone snails into genera [52], but it belongs to the subgenus *Cylinder* according to the molecular-based classification of cone snails into subgenera [53]. To resolve this controversy, we extracted and annotated by using BLAST searches for the 13 protein-coding and two ribosomal RNA (rRNA) mitochondrial genes from the assembled transcriptome (see below). These protein-coding and rRNA genes were aligned with orthologs from other cone snail mitogenomes (Supplementary Materials Table S4; [54]), using TranslatorX (http://translatorx.co.uk, accessed on 7 August 2021) [55] and MAFFT v.7 (EMBL-EBI, Hinxton, United Kingdom) [56], respectively. The individual alignments were combined into a concatenated dataset, and best-fit partitions and substitution models were inferred with PartitionFinder v.1 (https://www.robertlanfear.com/partitionfinder/, accessed on 7 August 2021) [57]. A maximum likelihood phylogenetic tree was reconstructed by using RaxML (https://cme.h-its.org/exelixis/web/software/raxml, accessed on 7 August 2021) v. 8.1.16 [58]. The reconstructed tree recovered the species here studied deeply nested within a clade including several species of the genus *Cylinder* (Supplementary Materials Figure S4), and therefore it was renamed as *Cylinder ammiralis* (following a generic classification), and this name is used throughout the study.

### 5.2. RNA Extraction and Sequencing

The venom gland was incubated in 300 µL of TRIzol (ThermoFisher Scientific, Waltham, MA, USA) and grinded with ceramic beads in a Precellys Evolution tissue homogenizer (Bertin Instruments, Montigny-le-Bretonneux, France). The solution was mixed with 60 µL of chloroform and centrifuged at 12,000× *g* for 15 min at 4 °C. The supernatant was recovered, mixed with one volume of isopropanol, and left overnight at −80 °C. Total RNA was purified by using the DirectZol miniprep kit (Zymo Research, Irvine, CA, USA), following manufacturer’s instructions.

Dual-index cDNA libraries were constructed by using the TruSeq RNA library Pep v2 (Illumina, San Diego, CA, USA), following manufacturer’s instructions. After the quality assessment of the libraries, these and other samples were pooled and split into several runs of paired-end sequencing (2 × 100 bp) in an Illumina HiSeq2500 (each pool divided into two flow cells), following standard procedures at Sistemas Genómicos (Valencia, Spain).

### 5.3. Transcriptome Assembly and Transcript Annotation

The raw reads were quality checked by using FastQC (https://www.bioinformatics.babraham.ac.uk/projects/fastqc/, accessed on 7 August 2021) [59] and assembled by using Trinity v.2.6.6 (https://github.com/trinityrnaseq/trinityrnaseq/releases/tag/Trinity-v2.6.6, accessed on 7 August 2021) [60] with default parameters (minimum contig length: 200; sequence identity threshold: 0.95), and the Trimmomatic option active (to remove Illumina adapters, trim low-quality regions and discard short sequences; SLIDINGWINDOW: 4; LEADING: 5; TRAILING: 5; MINLEN: 25). Identification of open-reading frames (ORFs) in the assembled transcriptome was performed with TransDecoder 2.0.1 (https://github.com/TransDecoder/TransDecoder, accessed on 7 August 2021) [61] and annotation with Trinotate 3.1.1 (https://github.com/Trinotate/Trinotate.github.io/wiki, accessed on 7 August 2021) [62].

The amino acid sequences of conotoxin precursors, hormones, and venom-related proteins were downloaded on 24 May 2020 from GenBank release 237, Uniprot release 2020_02, and ConoServer release 24 May 2020. The three databases were concatenated, and duplicates were removed. The resulting file was formatted as a BLAST+ database [63]. The transcripts encoding putative conotoxin precursors, hormones, and venom-related proteins of *C. ammiralis* were identified through BLASTX similarity searches of the assembled contigs against this database (e-value: 1 × 10^−5^). All contigs that were hit were manually inspected to remove false positives and to extract the correct CDS regions, creating a working list of proteins. Afterwards, several filtering steps were implemented. Assembly artifacts, such as chimaeras and sequences with low-coverage (<5 reads mapped), were detected in Tablet 1.19.09.03 [64] after mapping raw reads against all selected sequences, using Bowtie2 (http://bowtie-bio.sourceforge.net/bowtie2/index.shtml, accessed on 7 August 2021) [65], and then discarded. Sequences with low coverage in terminal positions were trimmed to avoid the inclusion of spurious variability in conotoxin isoforms. Duplicated and highly truncated (>55%) sequences were removed. The signal, propeptide, and mature regions of the remaining conotoxin precursors were identified by using the Conoprec tool (http://www.conoserver.org/?page=conoprec, accessed on 7 August 2021) [35]. Conotoxin precursors were assigned to the corresponding superfamilies based on the identity of the signal region, using an approximate threshold of 75% divergence [25]. Approximate relative expression levels of the transcripts from the different superfamilies were estimated by mapping clean reads back to assembled contigs. TPM (transcripts per kilobase million), which normalize for gene length and sequencing depth, were estimated with the RNA-Seq by Expectation Maximization (RSEM) package included in Trinity v.2.6.6 [60].

### 5.4. Venom Milking

A live gastropod snail (Nassariidae or Strombidae) was used to lure the cone snails in the aquarium and elicit the predatory behavior. Milking was conducted by using a microcentrifuge tube covered with parafilm and a piece of the snail to initiate stinging and collect the predation-evoked venom. To force a defense behavior, the cone snails were removed from the tank and disturbed by applying light pressure to the shell with long forceps. Once the proboscis was extended and stinging started, the defense-evoked venom was collected by using a microcentrifuge tube covered with parafilm. Several stings from the same individual were pooled for each of the venoms. The collected venoms were centrifuged, lyophilized, and stored at −20 °C.

### 5.5. LC–MS and Proteomic Analysis

The predation- and defense-evoked venom samples were subjected to liquid chromatography coupled with electrospray mass spectrometry (LC–ESI–MS) as previously described [6,16]. Briefly, ~500 μg of each venom was loaded onto a Kinetex C_18_ 100 Å column (2.1 mm × 150 mm, 3 µm) (Phenomenex, Torrance, CA, USA) fitted with a pre-column. The RP-UPLC runs were operated on an Acquity H-Class ultrahigh-performance liquid chromatography (UPLC) system (Waters, Corp., Milford, MA, USA) fitted with a UV detector (diode array detector) under the control of Waters MassLynx software version 4.1 (Waters, Corp., Milford, MA, USA). The samples were introduced into the mass spectrometer at a flow rate of 500 µL/min after their elution from the UPLC (gradient of 0–80% B 0.1% formic acid in acetonitrile in 80 min). Acquisitions were carried out over the range 50 Da to 1800 Da *m*/*z* every 0.1 s on a Synapt-G2-S high-definition MS system (Waters, Corp., Milford, MA, United States). Molecular masses were obtained by analyzing each peak from the total ion current (TIC) chromatogram with Waters Mass Lynx software (version 4.1) (Waters, Corp., Milford, MA, USA).

Next, the venom peptide/protein extracts were denatured, reduced, alkylated, and subjected to shotgun proteomics, as already described [66]. Briefly, each sample (~50 μg) was dissolved in 89 μL of triethylammonium bicarbonate (TEABC) 100 mM and reduced with dithiothreitol (DTT) 1 M for 30 min at 60 °C. Alkylation was performed with iodoacetamide (IAA) 0.5 M (incubation for 30 min in the dark). Samples were enzymatically digested by adding 2 μg of trypsin (Gold, Promega, Madison, WI, USA) in TEABC 100 mM and incubating overnight at 30 °C. After purification and concentration of the samples (OMIX Tips C_18_ reverse-phase resin, Agilent Technologies Inc., Santa Clara, CA, USA), peptides were dehydrated in a vacuum centrifuge and subjected to nano-flow liquid chromatography coupled to tandem mass spectrometry (NanoLC–MS/MS). Samples were resuspended in 20 μL formic acid (0.1%, buffer A), and 1 µL was loaded onto an analytical 25 cm reversed-phase column (75 mm inner diameter, Acclaim Pepmap 100^®^ C_18_, Thermo Fisher Scientific). Samples then were separated with an Ultimate 3000 RSLC system (Thermo Fisher Scientific, Waltham, MA, USA) coupled to a Q Exactive HF-X (Thermo Fisher Scientific, Waltham, MA, USA) via a nano-electrospray source, using a 123 min gradient of 6% to 40% of buffer B (80% ACN, 0.1% formic acid) and a flow rate of 300 nL/min. For the MS/MS analyses, full scans (375–1500 *m*/*z*) were acquired in the mass analyzer (Thermo Fisher Scientific, Waltham, MA, USA) with a 60,000 resolution at 200 *m*/*z*; 3 × 106 ions were accumulated within a maximum injection time of 60 ms and detected in the mass analyzer. The twelve most intense ions with charge states ≥2 were sequentially isolated to a target value of 1 × 105 with a maximum injection time of 45 ms and fragmented by higher-energy collisional dissociation (HCD) in the collision cell (normalized collision energy of 28%) and detected in the mass analyzer at 30,000 resolution.

### 5.6. Bioinformatic Integration of Proteomic and Transcriptomic Data

All the contigs assembled in the transcriptome analyses were translated into the six reading frames, creating the database used during the elucidation of the MS spectra. MS/MS spectra and transcriptomic sequences were matched by using the PEAKS Studio 8.5 software (Bioinformatics solutions, Waterloo, ON, Canada) with carbamidomethylation as fixed modification, while oxidation (M) was set as variable modifications, with maximum missed cleavages set at 3 for trypsin digestion. Parent mass was set to 5 ppm, while fragment mass error tolerance was 0.015 Da. Inaccurate proteins were filtered out by using a false discovery rate (FDR) of 1%, and there were two or more unique peptides. A −10lgP > 120 was used to estimate whether the detected proteins were identified by enough reliable peptides MS/MS spectra. Identification of additional mutations and sequence correction were performed with the Spider algorithm from PEAKS Studio software. This algorithm corrects the sequences stored in transcriptomic database with de novo sequences based on MS/MS spectra, which allowed us to identify post-translational modifications (PTMs) and mutations. Minimum ion intensity for mutation and PTMs was set to 5%, and ALC score ≥ 90 for de novo sequences, leading to low precursor mass error, in order to identify reliable PTMs and potential mutations.

### 5.7. Analysis of Venom Composition across Diets

The number of isoforms per conotoxin precursor superfamily was compared across cone snail species with different diets, using Illumina-based transcriptomic data. The following species represented molluscivorous cone snails: *Cylinder ammiralis* (this study), *Cylinder gloriamaris* [26], two *Cylinder victoriae* ([41] and ConoServer data i.e., the list of conotoxin precursors and conotoxins registered on ConoServer for this species), two *Darioconus episcopatus* ([67] and Conoserver data), and *Conus marmoreus* ([68] and ConoServer data). Vermivorous cone snail species were represented by *Africonus maioensis*, *Kalloconus trochulus*, and *Varioconus mercator* [11], *Dendroconus betulinus* [25], *Turriconus praecellens* [37], and *Calamiconus quercinus* [69]. Finally, three specimens of *Chelyconus ermineus* [15] and three of *Pionoconus magus* [27] were included to represent piscivorous cone snails (note that most previous transcriptomes reported for piscivorous species were based on 454 technology).

Once a list of contoxin precursor superfamilies was extracted for each specimen (Supplementary Materials Table S5), we tested for differences among diets by using the species as replicates in R [70] with the CRAN packages normtest [71] and dunn.test [72]. The normality of the data was checked by using the Shapiro–Wilk test, and once confirmed, the homocedasticity of the data was checked by using the Bartlett’s test, which is suitable for comparisons between more than two conditions with different sampling sizes. To identify those superfamilies whose abundances were significantly different among diets, we ran an ANOVA test for those samples that had passed both tests, or a Kruskal–Wallis test if one of them had failed. The post hoc analyses selected to assess the pairwise differences among diets were the Bonferroni correction (ANOVA) and the Dunn’s test (Kruskal–Wallis). Finally, the superfamilies from this dataset with variance zero were removed, and a principal component analysis (PCA) was run by using the prcomp function in R (R Core Team, Vienna, Austria) and plotted with ggplot2 (https://ggplot2.tidyverse.org, accessed on 7 August 2021) [73].

## Data Availability

The specimen of *C. ammiralis* analyzed in this study can be found in the Malacology collection of the National Museum of Natural Sciences (Madrid, Spain), under voucher number 15.05/87493. Raw reads were deposited at the SRA database with accession number SRR14921183, Bioproject PRJNA741614. Proteins identified in proteomic analyses were submitted to the NCBI database (accession numbers MZ484112-MZ484328). All protein sequences and their annotation can be assessed in the Supplementary Materials Tables S3 and S4.

## Supplementary Materials

The following are available online at https://www.mdpi.com/article/10.3390/toxins13090642/s1. Supplementary Materials Figure S1: Extension of Figure 1, including a second panel with all superfamilies grouped within the “Minor” category. Supplementary Materials Figure S2: Extension of Figure 1, including a second panel with details of all superfamilies showing a correlation between the number of peptides and the relative expression (measured in TPMs) between zero and four. Supplementary Materials Figure S3: PCA analysis of diet composition, measured as the number of conotoxin precursors identified for each superfamily. Supplementary Materials Figure S4: Phylogenetic ML tree based on complete mitochondrial genomes (concatenated 13 protein-coding genes plus two ribosomal RNA), bootstrap values are indicated above each node and scale bar indicates substitutions/site. The silhouette indicating the molluscivorous clade was downloaded from PhyloPic (T. Cunha; http://phylopic.org/, accessed on 7 August 2021). The pictures of the shells are from Alexander Medvedev (www.coneshells-am.ru, accessed on 7 August 2021). Supplementary Materials Table S1: List of conotoxin precursors, hormones, and venom-related proteins found in the venom gland transcriptome of *Cylinder ammiralis*. Supplementary Materials Table S2: List of the protein families found in the predation-evoked and defensive-evoked venoms, as well as in the venom gland transcriptome. Supplementary Materials Table S3: Summary of the statistical analyses comparing the abundance of each conotoxin precursor superfamily across diets. Supplementary Materials Table S4: Mitochondrial genomes used in the phylogenetic analyses. Supplementary Materials Table S5: Venom composition of all specimens used in the comparative analysis. Supplementary Materials File S1: Alignments of conotoxin precursors, hormones, and associated venom proteins of *Cylinder ammiralis* with homologues from other cone snail species. Supplementary Materials File S2: Alignments of the five conotoxin precursors of *Cylinder ammiralis* present in ConoServer with the closest sequences found in the transcriptome. Asterisks represent different amino acids in a given position. Supplementary Materials File S3: Fasta file containing the top 20 most expressed transcripts in the venom gland of *C. ammiralis*.
