# Supplementary material for: A Combined Transcriptomics and Proteomics Approach Reveals the Differences in the Predatory and Defensive Venoms of the Molluscivorous Cone Snail Cylinder ammiralis (Caenogastropoda: Conidae)
Source: Toxins (Basel). 2021 Sep 10;13(9):642. doi: 10.3390/toxins13090642 (PMC8472973; doi:10.3390/toxins13090642)

Supplementary Material Fig. S2 – Extension of the Fig. 2, including a second panel with details of all the superfamilies between zero and four

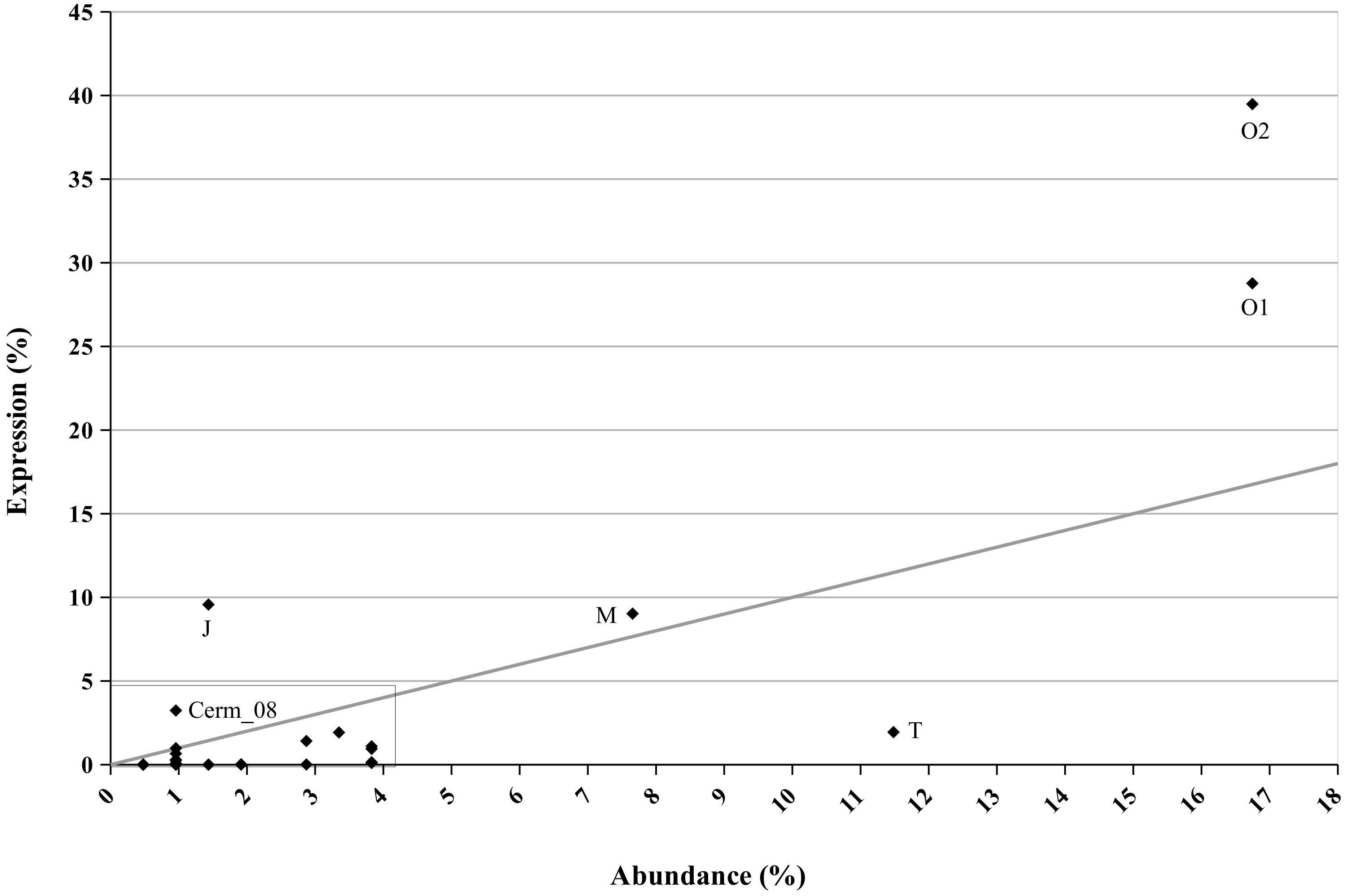

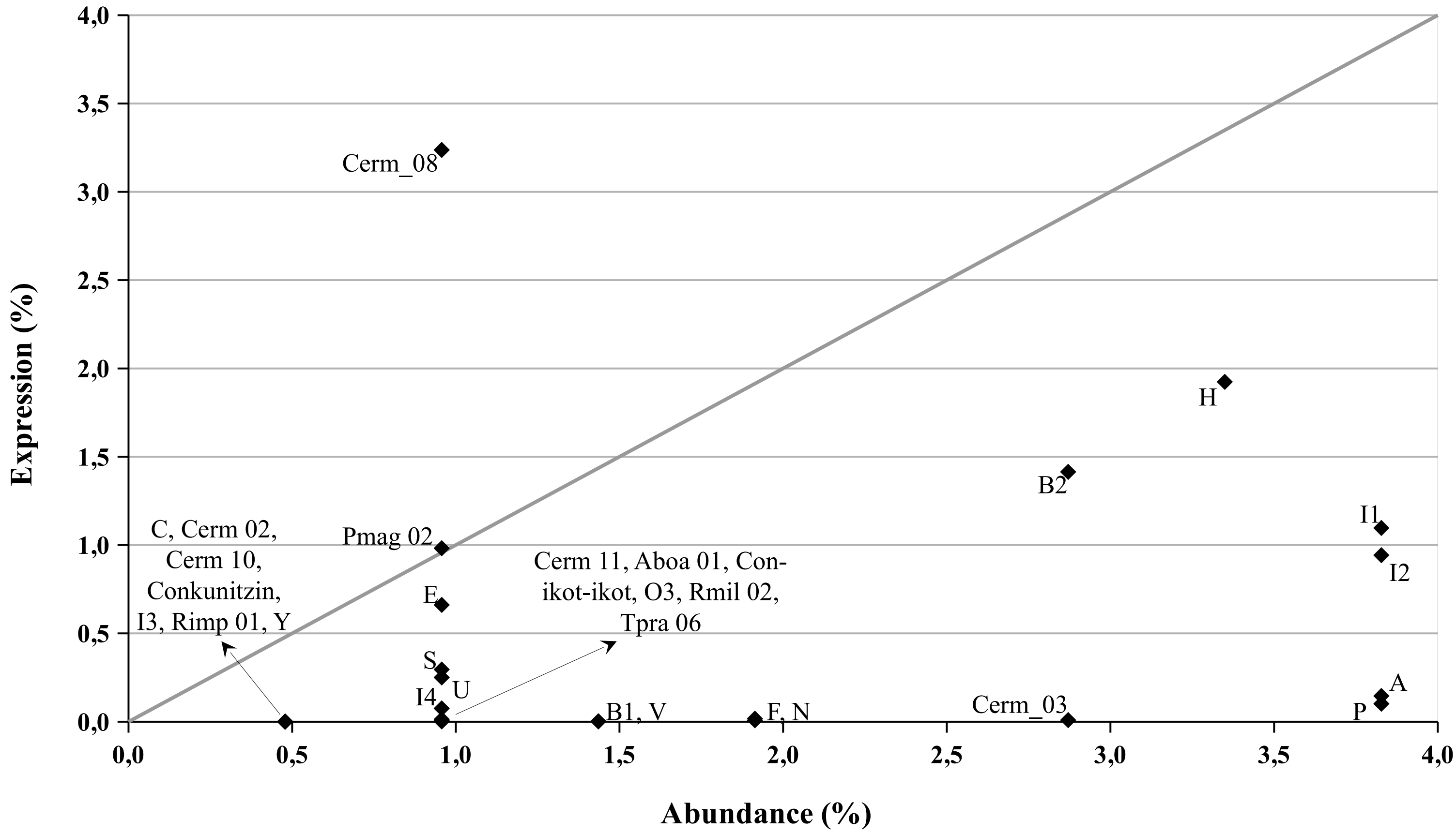

Supplement: Supplementary file 1 [file toxins-13-00642-s001.zip › Sup. Mat. Fig. S2 - Transcriptome-Abundance_vs_Expression.pdf]
