# Supplementary material for: A Combined Transcriptomics and Proteomics Approach Reveals the Differences in the Predatory and Defensive Venoms of the Molluscivorous Cone Snail Cylinder ammiralis (Caenogastropoda: Conidae)
Source: Toxins (Basel). 2021 Sep 10;13(9):642. doi: 10.3390/toxins13090642 (PMC8472973; doi:10.3390/toxins13090642)

Supplementary Material Figure S3 – PCA analysis of diet composition, measured as the number of conotoxins identified for each superfamily

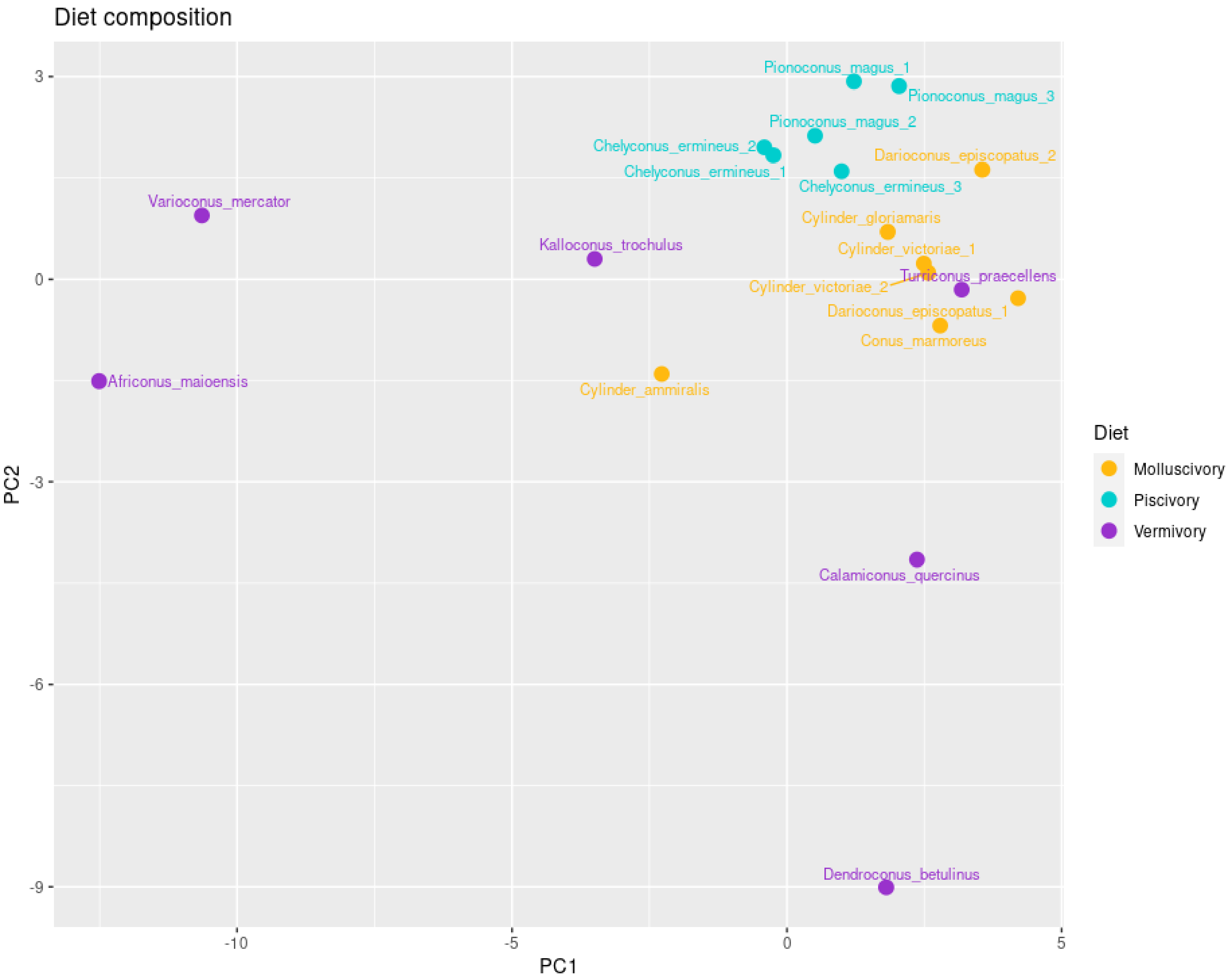

Supplement: Supplementary file 1 [file toxins-13-00642-s001.zip › Sup. Mat. Fig. S3 - PCA comparing diet composition accross diets.pdf]
